# Long-term efficacy and safety of cow’s milk anaphylaxis specific immunotherapy: Allergen unresponsiveness via the Tolerance Induction Program

**DOI:** 10.1016/j.jacig.2024.100285

**Published:** 2024-05-29

**Authors:** Inderpal Randhawa, Nathan Marsteller

**Affiliations:** a Food Allergy Institute, Long Beach, Calif; b Translational Pulmonary & Immunology Research Center, Long Beach, Calif

**Keywords:** Milk allergy, immunotherapy, machine learning

## Abstract

**Background:**

Tolerance Induction Program (TIP) immunotherapy applies machine learning contextualized on immunologic and food protein data sets. TIP has established efficacy toward peanut allergy. This form of treatment demonstrates equal efficacy toward cow’s milk anaphylaxis. TIP maintains remission outcomes defined as a minimum of 7 days of allergen unresponsiveness to high-dose protein exposures. Furthermore, remission patients openly consume unrestricted amounts of dairy protein.

**Objective:**

We sought to assess the rate of decline in specific IgE specific whole and component-resolved diagnostics following 1 year of TIP milk immunotherapy.

**Methods:**

The study comprised 214 cow milk anaphylactic children who underwent TIP at the Translational Pulmonary & Immunology Research Center/Food Allergy Institute. Postintervention changes in cow milk specific IgE, component-resolved diagnostics, and specific IgG4 were assessed.

**Results:**

After 1 year of 10-g dairy protein weekly sustained unresponsiveness, eosinophil count decreased from 558.38 to 409.26 cells/μL, the mean cow milk IgE decreased from 16.91 to 9.10 kU/L, the mean boiled cow milk IgE decreased from 12.89 to 6.03 kU/L, the mean Bos D4 decreased from 7.38 to 3.52 kU/L, the mean Bos D5 decreased from 6.79 to 3.16 kU/L, and the mean Bos D8 decreased from 13.55 to 6.62 kU/L. Adverse events were rare.

**Conclusions:**

TIP cow milk immunotherapy significantly reduced cow milk specific IgE and component-resolved diagnostics while increasing specific IgG4 in cow milk anaphylactic children. TIP demonstrates safety and clinical efficacy in cow milk anaphylaxis treatment.

Cow’s milk allergy is a leading food allergy in early childhood, with estimated prevalence between 0.5% and 3% until age 1 year.^1^ Cow’s milk allergy has increased in incidence over the last decade. Although commonly outgrown by age 3 years, cow’s milk allergy now presents into young adulthood.^2^ Anaphylaxis fatality rates remain between 0.65% and 2%, with food allergy a leading trigger.^3^ Despite avoidance practices, the current disease state of milk allergy led to various treatment approaches.

Baked cow’s milk introduction in the format of a “ladder” has been published.^4^ The approach to baked milk immunotherapy is variable, with tolerance rates between 50% and 83% among multiple small studies.^5^ The “ladder” involves escalation of the baked good intake over time. Despite evidence of cow’s milk tolerance over time, the “ladder” denotes a significant adverse event rate including anaphylaxis to the baked milk dose.^6^ Oral immunotherapy (OIT) using diluted, uncooked cow’s milk has been described.^7^ Few studies promote long-term open consumption of cow’s milk with OIT. However, the adverse event rate including nonanaphylactic and anaphylactic reactions remains elevated. De Schryver et al^8^ reported a milk OIT cohort mean anaphylactic reaction rate of 6 events per patient per year. Furthermore, 27% of the cohort discontinued treatment in total. Aiming to mitigate adverse events, the cow’s milk patch epicutaneous immunotherapy approach remains under clinical trials. No published data to date demonstrate high efficacy cow’s milk immunotherapy with minimal adverse events.

The Tolerance Induction Program (TIP) involves the use of machine learning and boosting analytics across databases of immunobiology (allergic and tolerance markers) and biosimilar proteins. Biosimilar proteins in cow’s milk allergy have been well described in agricultural science.^8^ Mammalian proteins biosimilar to cow’s milk include camel, mare, donkey, goat, and buffalo milk proteins. Casein and whey derivatives have been characterized with biosimilarity across protein sequence and protein content.^9^, ^10^, ^11^ Cross-sensitization of mammalian milks in cow’s milk allergy demonstrates associated IgE binding.^12^ Camel milk and donkey milk studies in cow milk allergy cohorts reflect tolerability.^13^, ^14^, ^15^ However, the molecular effect of the intervention was not reported. TIP machine learning training data were developed in a continuous model across enrolled patients. Accumulation of data across specific IgE subdivision served as the basis of data organization. Multiple support vector machines and boosting algorithms developed dose vectors of individual mammalian milks based on casein and whey content. The initial model was further assessed with sequential patient data over time. A Bayesian method was then used to optimize dose vector use by mammalian protein class in sequence until cow milk treatment commenced. Testing data were used on subsequent patient data to provide the final hybrid machine learning prediction algorithm.

Allergen unresponsiveness in food allergy is not clearly defined. A proposed stringent definition by Berin and Mayer^16^ describes sustained unresponsiveness as clinical nonresponsiveness to food allergen after complete discontinuation of therapy. If regular antigen-specific immunotherapy maintenance is continued, the immunologic state is deemed desensitized.^16^ However, complete discontinuation of food allergen after immunotherapy has never been reported. Specific to cow milk allergy, Manabe et al^17^ defined sustained unresponsiveness as a 2-week period between low-dose maintenance exposure.^17^ Suárez-Fariñas et al^18^ further described 23 milk allergy patients who underwent OIT with omalizumab following an 8-week period of sustained unresponsiveness to a high-dose 10-g cow milk challenge. However, only 40% of the small cohort was able to achieve this definition of sustained unresponsiveness.^18^ TIP patient outcomes have defined allergen unresponsiveness as a 7-day period of nonexposure followed by high-dose allergen exposure.^19^ Unique to TIP, allergen unresponsiveness is monitored for years and free, unrestricted consumption of dairy is allowed.

To date, cow’s milk OIT studies consist of small cohorts with short study periods. End points of such studies focus on maximum number of milliliters tolerated for a fixed time period. The concept of allergen unresponsiveness in milk oral immunotherapy has been reported.^17^ Reported outcomes were based on small patient surveys. Here, we report the largest cow’s milk anaphylactic cohort to undergo immunotherapy. It is the first study using the TIP machine learning and artificial intelligence system specific to dairy anaphylaxis. TIP treatment achieved 1-week allergen unresponsiveness in this cohort. The comprehensive immune diagnostic and clinical outcomes of the patient-specific treatment are further detailed.

## Methods

### Patients

This was a prospective, descriptive study in cow milk allergic children aged 1 to 21 years who enrolled in TIP therapy at the Translational Pulmonary and Immunology Research Center (TPIRC) from January 2019 to July 2021. Participant inclusion criteria required at least 1 anaphylactic episode of clinical history of grade 2 anaphylaxis verified by hospital medical record within 12 months of study entry. Additional criteria included a cow milk skin prick test wheal diameter greater than 3 mm or specific IgE or cow milk component Bos d resolved diagnostics greater than 0.1 kU/L. No food challenge to cow milk was required. Each parent and patient were told that the standard of care for cow milk allergy was avoidance and preparedness for treating reactions. Informed consent and assent was obtained from all study participants under Good Clinical Practice in clinical research and according to established ethical and regulatory standards of the Advarra Institutional Review Board (IRB #PRO00043361, Columbia, Md).

### Study process and design

TIP involves use of machine learning and boosting analytics assessing hundreds of proallergic markers (skin prick testing, component-resolved diagnostics, Immuncap specific IgE, histamine release assay, peripheral eosinophil count) as well as tolerance markers (cytokine profiles [IL-4, IL-5, IL-10, IL-13], IgG4 specifics, total IgG4, total IgG) across collected patient data since 2007. Allergic and tolerance markers are laboratory analytes via Immunocap or laboratory-developed tests (TPIRC Diagnostics, Long Beach, Calif) to specific allergens categorized as animal proteins (fowl, fish, epidermal, meat, milk, eggs, shellfish) as well as plant proteins (pollens, grains, seeds, and nuts). All patients undergo the same range of diagnostic testing as part of machine learning database development. IgG4 data are reported as μg/mL (range, 0-30). IgE data are reported as kU/L.The study was conducted under the supervision of Advarra Institutional Review Board under Protocol 00040982. All patients gave written informed consent and/or assent per Institutional Review Board guidelines.

Currently, the analytics process organizes an incoming patient’s specific markers against the molecular groupings of the database. Once analyzed, the patient’s markers are assessed into an endotype solely on the basis of applied mathematical arrays. Endotype 1 requires clinical and laboratory markers of anaphylaxis to both plant and animal proteins. Endotype 2 requires a total IgG 1 SD below normal (age specific). Endotype 3 requires clinical and laboratory markers of anaphylaxis to only animal proteins. Endotype 4 requires the total IgE level less than 110 kU/L. Endotype 4 requires a pathologic diagnosis of eosinophilic esophagitis on biopsy at more than 15 eosinophils/HPF.

Mammalian milk proteins used in TIP include camel milk, mare milk, donkey milk, sheep milk, goat milk, and cow milk. Mammalian milks are organized in databases on the total whey and casein derivative proteins in an uncooked and fully denatured state. Fully denatured mammalian milk comprises boiled milk at 95ºC for 3 minutes. Cross-contamination of mammalian protein product sources was limited on the basis of specific manufactured product requirements.

Mammalian milk proteins were sourced from specific commercial vendors. Study participants were provided the milk proteins directly from the TPIRC for treatment and maintenance cycles. All proteins underwent sample testing of dairy protein content through Kjeldahl methods by Eurofins (Fresno, Calif). Camel milk protein 7 g/25 g mass was sourced from Desert Farms (Irvine, Calif). Mare milk protein 5.4 g/20 g mass was sourced from Saumal (Karaganda, Khazikhstan). Donkey milk protein 4 g/24 g mass was sourced from Montebaducco (Quattro Castella, Italy). Sheep milk protein 31 g/100 g mass was sourced from Spring Sheep (Auckland, New Zealand). Goat milk protein 7 g/28 g mass was sourced from Meyenberg (Salinas, Calif). Cow’s milk protein 13 g/8 oz volume was sourced from Fairlife Milk (Chicago, Ill).

TIP machine learning prioritization, immunotherapy sequence, and cycle length of mammalian milk protein target ingestion amounts are based on the machine learning boosting algorithms to safely cross-match and condition exposure based on whey and casein derivative content. Biosimilar mammalian proteins used included camel, mare, donkey, sheep, and goat milk. Camel milk target total protein ranged from 0.25 to 1.5 g. Mare milk target total protein ranged from 0.3 to 1.4 g. Donkey milk target total protein ranged from 0.3 to 2.0 g. Sheep milk target total protein ranged from 0.5 to 3 g. Goat milk target total protein ranged from 0.3 to 2.0 g. Cow milk target total protein ranged from 0.3 to 4.0 g. Mammalian milk proteins were consumed in sequence as immunotherapy. Completion of 1 cycle of protein moved the protein to maintenance for 1 subsequent cycle. Cycles ranged from 6 to 8 weeks.

Patients received start dose exposure in the TIP clinical center comprising complete cardiac and respiratory monitoring. Subsequent dosing occurred at home. Updosing occurred at home on a weekly or biweekly basis designed by machine learning analytics. Once specific target protein vectors were reached, patients returned to the TIP clinical center for a complete target protein challenge followed by an introduction of the next cycle protein. After passing the 4 g total protein cow milk challenge, patients maintained 4 g cow milk protein daily for 4 months. The patients then underwent a 10-g cow milk protein challenge (6 oz of ultrafiltered cow milk) followed by weekly maintenance of 10 g once weekly for at least 1 year of follow-up. At this state of allergen unresponsiveness, patients were allowed to consume any amount of dairy protein without restriction. The protocol permitted adjustments to the weekly home updosing schedule as needed; for example, temporary dose stoppages were allowed while subjects were suffering from symptoms of an upper respiratory tract infection or influenza, or during menses. Subjects were cautioned against activities likely to increase reactivity within 1 hour after dosing. After 1 year of allergen unresponsiveness, patients underwent repeat marker analysis. The exposure rate as described served as a template of allergen unresponsiveness where the amount of exposure between dose exposure and nonexposure is reflective of real-world dietary risk exposure.

No patients were on omalizumab before or during treatment. No patients were on any form of systemic steroids. Only as-needed antihistamines were used by patient preference for seasonal rhinitis symptoms in the first year of allergen unresponsiveness.

### Assessment of clinical efficacy and adverse events

TPIRC’s food allergy branch in Long Beach, Calif, performs extensive Immunocap and skin prick testing to dairy in addition to measurement of other entry diagnostic markers. Specific IgE antibodies to mammalian milk proteins were measured using the ImmunoCap (Phadia, Kalamazoo, Mich) fluorescent enzyme immunoassay in our laboratory. Additional measures collected at baseline and 12-month follow-up included cow milk proallergy markers Bos d 4, Bos d 5, Bos d 8, specific IgE, antiallergy marker IgG4-cow milk, and associated markers serum eosinophils and total IgE. Adverse events were documented using a 24/7 on call phone and portal system that records every adverse event. Patient diaries were not used. However, patient intake assessments were taken during every visit to ensure compliance and provide a secondary source of adverse event data.

### Statistical methods

Considering substantial departures from both the normality and absence of outliers’ assumption of all 8 independent-samples *t* tests, these analyses were deemed unreliable to assess the differences in the pretreatment and posttreatment IgE values. Thus, the Wilcoxon signed-rank test was used as the nonparametric counterpart to the dependent-samples *t* test to assess the difference between pretreatment and posttreatment values. The second portion of analysis involved mixed-effects modeling using a subject-specific approach to patterns in the changes in the pretreatment to posttreatment values in Bos D5, Bos D8, and milk IgG4. Before running the analysis, a grouping variable was created that assigned patients to 4 groups on the basis of their preintervention. A random intercept and fixed slope model and a random intercept and random slope model were used with a gamma specification family and with a log link function.

## Results

Data from a total of 214 patients were included for analyses. All 214 patients completed follow-up of the study. All patients in the cohort had reported a recent severe anaphylactic reaction to cow’s milk within 12 months of enrollment. The age of the patients at baseline ranged from 1 to 19 years and with an average of 5.6 years (median, 8.7 years). The mean duration of dairy immunotherapy was 21 months (639.2 days). The mean measured period of weekly allergen unresponsiveness was 12.6 months (383.2 days). Of the 214 patients, 33.6% (n = 72) were female and 66.4% (n = 142) were male. The frequencies of types 1, 2, 3, 4, and 5 endotypes were 57 (26.6%), 124 (57.9%), 6 (2.8%), 4 (1.9%), and 2 (0.9%), respectively, and 21 patients were nontypable.

Table I provides descriptive statistics for the variables of the study. From pretreatment to posttreatment, the mean total IgE increased from 573.93 to 784.56, and the mean milk IgG4 increased from 3.08 to 7.17. However, the mean eosinophil count decreased from 558.38 to 409.26, the mean cow milk IgE decreased from 16.91 to 9.10, the mean boiled cow milk IgE decreased from 12.89 to 6.03, the mean Bos D4 decreased from 7.38 to 3.52, the mean Bos D5 decreased from 6.79 to 3.16, and the mean Bos D8 decreased from 13.55 to 6.62.

**TABLE I:** Descriptive statistics for the study variables

Variable	n	Minimum	Maximum	Median	IQR	Mean ± SD
Pre–total IgE	206	7.56	5960	321.50	546.50	573.93 ± 788.09
Post–total IgE	206	18.2	13510	389.00	712.50	784.56 ± 1284.05
Preeosinophil	208	10	4241	370.00	504.50	558.38 ± 590.28
Posteosinophil	208	10	2530	355.00	360.00	409.26 ± 298.09
Pre–cow milk IgE	141	0	101	2.71	15.71	16.91 ± 29.25
Post–cow milk IgE	141	0	100	1.59	8.20	9.10 ± 17.61
Pre–boiled cow milk IgE	213	0	101	1.90	10.77	12.89 ± 25.04
Post–boiled cow milk IgE	213	0	100	0.80	4.01	6.03 ± 14.25
Pre–Bos D4	214	0	100	0.52	4.35	7.38 ± 17.85
Post–Bos D4	214	0	69.7	0.28	2.50	3.52 ± 8.91
Pre–Bos D5	214	0	100	0.47	3.05	6.79 ± 17.95
Post–Bos D5	214	0	87.3	0.23	1.36	3.16 ± 9.58
Pre–Bos D8	214	0	101	1.25	9.80	13.55 ± 26.46
Post–Bos D8	214	0	100	0.61	3.18	6.62 ± 16.87
Premilk IgG4	214	0	30	0.34	1.90	3.08 ± 6.46
Postmilk IgG4	214	0	30	1.72	7.74	7.17 ± 10.30

*IQR*, Interquartile range.

**Fig 1:**
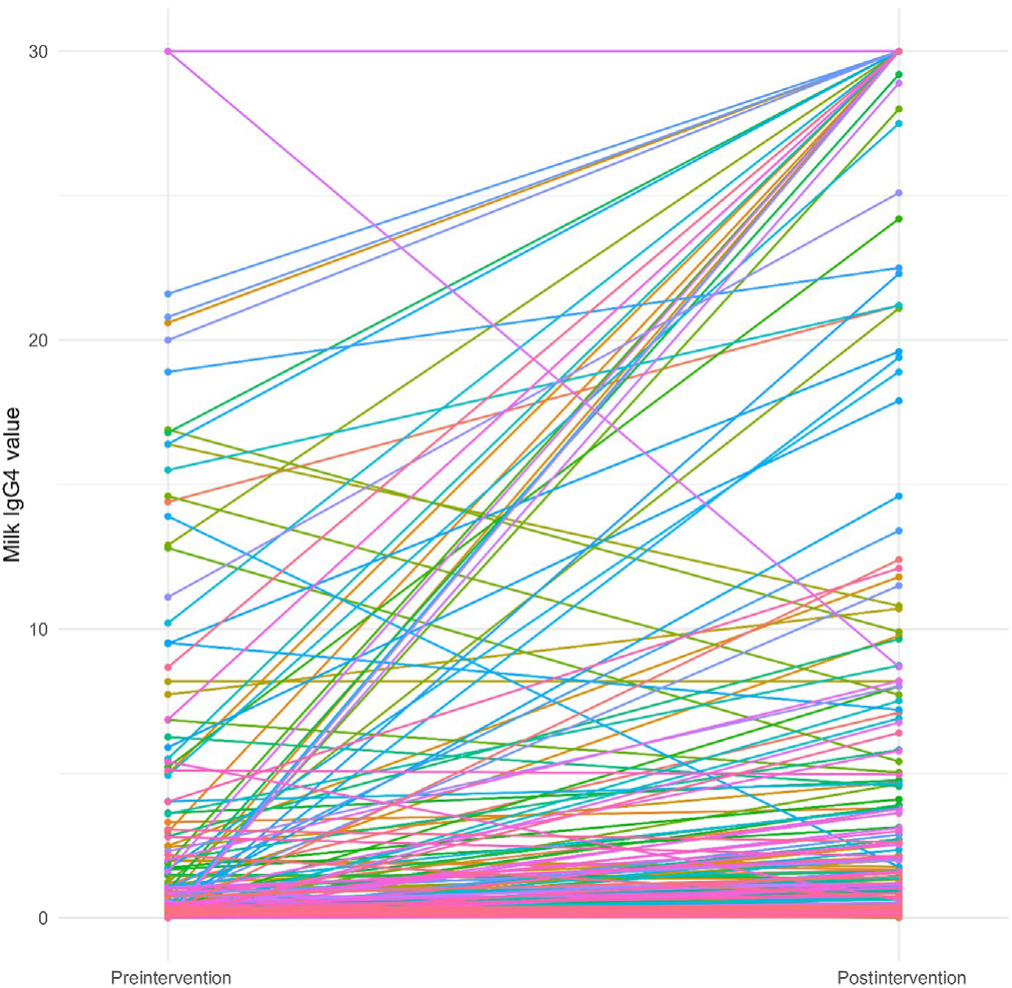
Changes in milk IgG4 values from preintervention to postintervention for individual patients. IgG4 data are reported as μg/mL (range, 0-30).

**Fig 2:**
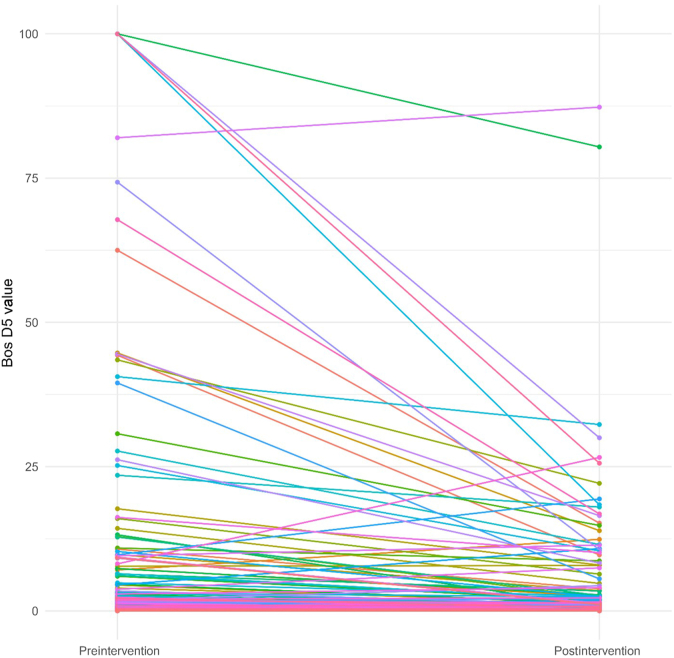
Changes in Bos D4 values from preintervention to postintervention for individual patients. IgE data are reported as kU/L (range, 0-100).

**Fig 3:**
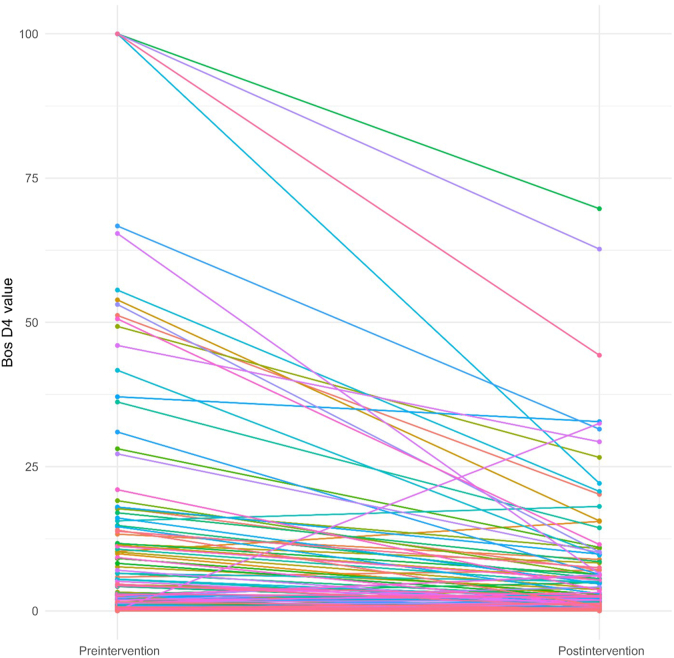
Changes in Bos D5 values from preintervention to postintervention for individual patients. IgE data are reported as kU/L (range, 0-100).

The results of the Wilcoxon signed-rank test are summarized in Table II. These results indicated that there were significant differences between preintervention and postintervention value for all 8 IgEs (*P* < .05). The median of total IgE increased from 321.50 to 389.00. The median of milk IgG4 increased from 0.34 to 1.72 (Fig 1). Both increases in the medians were statistically significant. The median of eosinophil decreased from 370 to 355, the median of cow milk IgE decreased from 2.71 to 1.59, the median of boiled cow milk IgE decreased from 1.90 to 0.80, the median of Bos D4 decreased from 0.52 to 0.28 (Fig 2), the median of Bos D5 decreased from 0.47 to 0.23 (Fig 3), and the median of Bos D8 decreased from 1.25 to 0.61. Based on the results of the Wilcoxon signed-rank tests, all these decreases in the medians were statistically significant.

**TABLE II:** Assessing differences between preintervention and postintervention IgE values

Variable	N	Wilcoxon test statistic	*P* value
Total IgE	206	7,816.5	.001
Eosinophil	208	13,172	.001
Cow milk IgE	141	7,127	<.001
Boiled cow milk IgE	213	16,341	<.001
Bos D4	214	11,997	<.001
Bos D5	214	12,070	<.001
Bos D8	214	15,540	<.001
Milk IgG4	214	2,839.5	<.001

### Adverse events

The cohort completed 1046 oral food challenges (OFCs) of mammalian milk proteins during the study period. OFCs included 260 camel milk, 154 mare milk, 114 donkey milk, 110 sheep milk, 194 goat milk, and 214 cow milk. All compliance and adverse events are recorded during food challenge visits by direct patient reporting or direct clinical observation. The cohort completed 54,397 oral home doses of mammalian milk proteins during the study. Among OFC and home doses, there were a total of 6 (0.01%) treatment-associated epinephrine-requiring events during the total cohort treatment time. Non–epinephrine-requiring reactions during the total treatment period were 72 events (1.3%). Of the 72 events, 22 events required no medical intervention, 31 events required diphenhydramine, and 19 events required diphenhydramine plus additional medications such as H2 blockade medications and prednisolone. Of the 72 events, 12 occurred during OFCs. The total number of emergency room visits due to adverse events was 5. No emergency rooms visits occurred from adverse events of OFCs. Zero treatment-associated epinephrine-requiring events were recorded during the 1 year of weekly allergen unresponsiveness.

## Discussion

This is the first study in food allergy to monitor a pediatric cow’s milk allergy cohort of substantial size across years of active treatment. It is the first publication using the TIP machine learning and artificial intelligence system specific to dairy anaphylaxis. Previous cow’s milk oral immunotherapy studies with clinical trial design have demonstrated successful desensitization.^19^ However, no long-term clinical trial outcomes achieved unlimited intake of cow’s milk. The disease remission milestone reached in this study reflects 1 week of allergen unresponsiveness to cow’s milk with subsequent no limit to specific dairy allergen intake. Furthermore, the continued downregulation of IgE specific cow’s milk components in this cohort yields promise to a long-term state of anaphylactic disease abatement.

This study aimed to ascertain TIP-induced differences between specific IgE levels pretreatment and posttreatment. Data assessed the changes in the pretreatment and posttreatment values for total IgE, eosinophil count, cow milk IgE, boiled cow milk IgE, Bos D4, Bos D5, Bos D8, and milk IgG4. Posttreatment data are reflective of weekly, high-dose cow milk protein of 10 g at 7-day allergen unresponsiveness intervals. These results indicated that after treatment statistically significant increases in the total IgE and milk IgG4 values were present. However, significant decreases were demonstrated in total eosinophil count, specific cow milk IgE, specific boiled cow milk IgE, Bos D4 IgE, Bos D5 IgE, and Bos D8 IgE.

TIP results specific to milk proteins are similar to previously published studies in peanut allergy.^20^ TIP uses applied math targeting evolutionary protein databases and immunobiology databases to assess the specific risk profile of a food allergy patient. The machine learning algorithms for TIP have been published.^21^ The risk profile designs the specific mammalian protein sequence, protein type, protein amount, and vector dose rate for each patient. This biosimilar protein immunotherapy model is a key component to the clinical goal of achieving high-dose protein allergen unresponsiveness. The machine learning algorithms follow patient data for years. Although not presented in this study, TIP patients reduce the frequency of high-dose 10-g weekly cow milk protein to biweekly and monthly doses based on further reduction in cow milk specific IgE markers and clinical tolerance of dairy intake.

IgE and IgG4 markers in cow’s milk allergy have been characterized in natural tolerance.^22^ Reduction in specific IgE is noted among persistence versus tolerance of egg allergy.^23^ Previous OIT studies characterize the molecular response to food protein immunotherapy as a short-term increase in specific IgG4 with a variable reduction in specific IgE.^24^ Biosimilar protein immunotherapy through our TIP cohort resulted in a modest yet statistically significant increase in specific IgG4. However, the continued reduction in all dairy protein specific IgE parameters during weekly high-dose dairy applications is significant in the field of allergy immunotherapy. Of note, the treatment effect in this large cohort is tethered to an exceptionally low total adverse event rate and minimal epinephrine use.

Limitations of our study are specific. The cohort skews toward the pediatric male population. The lack of a food challenge at the initiation of treatment is a limiting factor despite strong clinical history of anaphylaxis within 12 months of enrollment. Persistence of cow’s milk allergy in younger patients of the cohort is a question. Natural tolerance to cow’s milk in those younger than 3 years is well described with age implications.^25^ However, given the median age of the cohort was 8.7 years, the effect of possible nonpersistent allergy is less likely. The trajectory of a small portion of the cohort at enrollment may have been toward natural tolerance. The degree of compliance during the first year of allergen unresponsiveness was difficult to ascertain by patient survey. Nevertheless, variable consumption of dairy during this time only reflects the stability of allergen unresponsiveness. Allergen unresponsiveness in our cohort may reflect a prolonged form of desensitization. However, such forms of desensitization have not been reported at high-dose protein exposure intervals. In addition, the TIP method allows for open consumption of dairy protein, which is rare in food allergy immunotherapy outcomes.

TIP cow milk immunotherapy significantly reduced cow milk specific IgE and component-resolved diagnostic assays while increasing specific IgG4 in cow milk anaphylactic children. TIP demonstrates remarkable safety and clinical efficacy in cow milk anaphylaxis treatment. Mathematical models and machine learning have a growing presence in modern medicine. Head-to-head trials of TIP versus traditional OIT are indicated to further differentiate the 2 modalities of immunotherapy.

**Clinical implications**

TIP innovative cow’s milk immunotherapy demonstrates safety, efficacy, and long-term remission in cow’s milk anaphylaxis.

## Disclosure statement

This study was supported by the Translational Pulmonary & Immunology Research Center.

Disclosure of potential conflict of interest: The authors declare that they have no relevant conflicts of interest.
